# Feet and legs malformation in Nellore cattle: genetic analysis and prioritization of GWAS results

**DOI:** 10.3389/fgene.2023.1118308

**Published:** 2023-08-16

**Authors:** Thales de Lima Silva, Cedric Gondro, Pablo Augusto de Souza Fonseca, Delvan Alves da Silva, Giovana Vargas, Haroldo Henrique de Rezende Neves, Ivan Carvalho Filho, Caio de Souza Teixeira, Lucia Galvão de Albuquerque, Roberto Carvalheiro

**Affiliations:** ^1^ Department of Animal Science, School of Agricultural and Veterinarian Sciences, São Paulo State University (Unesp), Jaboticabal, SP, Brazil; ^2^ Department of Animal Science, College of Agriculture and Natural Resources, Michigan State University, East Lansing, MI, United States; ^3^ Centre for Genetic Improvement of Livestock, Department of Animal Biosciences, University of Guelph, Guelph, ON, Canada; ^4^ Department of Animal Science, Viçosa Federal University, Viçosa, Brazil; ^5^ GenSys Associated Consultants, Porto Alegre, Brazil; ^6^ Researcher at National Council for Scientific and Technological Development (CNPq), Brasília, Brazil

**Keywords:** *Bos taurus indicus*, candidate genes, functional trait, locomotion, welfare

## Abstract

Beef cattle affected by feet and legs malformations (FLM) cannot perform their productive and reproductive functions satisfactorily, resulting in significant economic losses. Accelerated weight gain in young animals due to increased fat deposition can lead to ligaments, tendon and joint strain and promote gene expression patterns that lead to changes in the normal architecture of the feet and legs. The possible correlated response in the FLM due to yearling weight (YW) selection suggest that this second trait could be used as an indirect selection criterion. Therefore, FLM breeding values and the genetic correlation between FLM and yearling weight (YW) were estimated for 295,031 Nellore animals by fitting a linear-threshold model in a Bayesian approach. A genome-wide association study was performed to identify genomic windows and positional candidate genes associated with FLM. The effects of single nucleotide polymorphisms (SNPs) on FLM phenotypes (affected or unaffected) were estimated using the weighted single-step genomic BLUP method, based on genotypes of 12,537 animals for 461,057 SNPs. Twelve non-overlapping windows of 20 adjacent SNPs explaining more than 1% of the additive genetic variance were selected for candidate gene annotation. Functional and gene prioritization analysis of candidate genes identified six genes (*ATG7*, *EXT1*, *ITGA1*, *PPARD*, *SCUBE3*, and *SHOX*) that may play a role in FLM expression due to their known role in skeletal muscle development, aberrant bone growth, lipid metabolism, intramuscular fat deposition and skeletogenesis. Identifying genes linked to foot and leg malformations enables selective breeding for healthier herds by reducing the occurrence of these conditions. Genetic markers can be used to develop tests that identify carriers of these mutations, assisting breeders in making informed breeding decisions to minimize the incidence of malformations in future generations, resulting in greater productivity and animal welfare.

## 1 Introduction

Brazil is the largest exporter of beef in the world in millions of tons (14.4% of the international market) and ranks third in value (US$ 7 billion) (Aragão and Contini, 2021). The Nelore is the main breed (80%) of the national herd and the Special Certificate of Identification and Production (CEIP) (MAPA, 2006) allows 20%–30% of the best bulls of each year to be certified as genetically superior. This guarantees a differentiated market value for the animals and their products as well as tax benefits when marketing them. CEIP is also a requirement to register animals as semen donors in the Breed Registration Service. However, animals of high genetic value and CEIP candidates are disqualified and slaughtered when affected by some morphological disorder, even though they have great genetic value for other traits of economic importance (e.g., reproductive traits, growth, and carcass quality). This leads to a decrease in efficiency in meat production systems due to the deficit of superior bulls from breeding programs to carry out natural mating in commercial herds (Rosa et al., 2021).

Animals affected by feet and legs malformations cannot perform their productive and reproductive functions satisfactorily, resulting in significant economic losses. The term “limb deformities” usually refers to defects in the legs and feet caused by the most varied etiological agents in different species of production animals (Rahe et al., 2022; Schwertz et al., 2023). Here we adopted the term feet and legs malformation (FLM) as a binary functional trait (having or not having defect) previously reported in the literature (Vargas et al., 2017; 2018), with a heritability of 0.18 (SE = 0.04) and an incidence of approximately 5% in Nellore cattle (Vargas et al., 2017). Efforts to reduce the incidence of FLM in Nellore cattle must begin by studying the degree of genetic variability in the population and possible genetic associations with other traits in the selection process. The genetic correlation between FLM and yearling weight (YW) (Vargas et al., 2017) suggests the possibility of using the second trait as an indirect selection criterion but needs to be recalculated due to the increased number of records available for FLM in this Nellore population.

Identification of significant regions in the bovine genome and candidate genes associated with FLM can also help to understand the biology behind the phenotype. In this way, Genome-wide Association Studies (GWAS), using phenotype, genotype, and pedigree data through the weighted single step genomic best linear unbiased predictor (WssGBLUP) method (Legarra et al., 2009) enables the identification of genomic regions that may be associated with the trait of interest. In a functional enrichment analysis, such as the one performed by Vargas et al. (2018), SNP windows that explain the highest proportion of additive variance are investigated in a database such as the Genome Data Viewer of the National Center for Biotechnology Information (NCBI, 2022) looking for genes located in these genomic regions. The presence of adjacent window genes of up to 1 Mb upstream and downstream of the analysed windows is also evaluated, as their effect can be captured by adjacent SNPs due to linkage disequilibrium (LD). Then articles published in indexed scientific journals are considered to support with biological evidence the potential role of genes located in these windows on the trait. However, the identification of functional candidate genes also can be performed using a systems biology approach, where genes shared between traits and studies are evaluated by a guilt by association gene prioritization (GUILDify and ToppGene software) in order to identify the best functional candidates (Fonseca et al., 2018).

This work aims to better understand the molecular mechanisms underlying FLM and provide subsidies that help to reduce the incidence of this problem in breeding programs for Nellore cattle, contributing to improve their production and reproductive efficiency. Vargas et al. (2017) defined the FLM genetic basis and Vargas et al. (2018) associated FLM with SNPs and performed a gene enrichment analysis. These previous analyses were updated using now a new genomic reference map (ARS-UCD1.2, Hu et al., 2022) and a larger dataset (295,031 phenotypes and 12,537 genotypes), in addition to apply a new methodology for the functional enrichment analysis of positional candidate genes (Fonseca et al., 2018). The objectives of this work were: 1) to re-estimate the genetic parameters of FLM, including genetic correlation between FLM and yearling weight (YW); 2) estimate genetic trends for these traits in Nellore cattle; 3) to find windows of adjacent SNPs significantly associated with FLM using the WssGBLUP methodology, and 4) to identify candidate genes supported by functional evidence with a potential role in the incidence of FLM.

## 2 Materials and methods

Approval from the Welfare and Animal Use Committee was not required for this study because the data was obtained from an existing database of phenotypic and genotypic records from Nellore cattle. Previous studies using these data also did not need this approval.

### 2.1 Population structure and phenotypic data

A final dataset of 295,031 Nellore cattle from the Nellore Alliance (GenSys, 2019), all with phenotypic information, collected between 2001 and 2017 was used in this study. Feet and legs malformation were evaluated by trained technicians which assigned binary scores (1 for affected animals and 0 for unaffected) to the overall structure of feet and legs at yearling (Vargas et al., 2018). Contemporary groups (CG) were defined considering the effects of herd, year and season of birth, sex, management group at weaning and yearling, date of measurement at yearling. Only data from CG with more than 10 records, variability in the trait and at least 10 genetic links to other CG were kept for the analyses. Connectedness among CG was checked using AMC software (Roso and Schenkel, 2006) with default parameters. As a result of the use of lots of multiple sires in the breeding season on part of the farms that make up the database, 31% (90,500) of the animals had the father information as unknown.

### 2.2 Genetic parameter estimates

After checking consistency and editing data, estimates of variance components and estimated breeding values (EBV) were obtained for each animal by Bayesian inference using THRGIBBS1F90 software (Misztal et al., 2014). To evaluate a possible correlated selection response of YW in FLM, a linear-threshold animal model was fitted to the two-trait analysis. This statistical model was also used by Vargas et al. (2017) and can be written in matrix notation as:

$$l_{i}=X\beta_{i}+Za_{i}+e_{i}$$

where $l_{i}$ is the vector of observations (or liabilities) for the *i*th trait, $\beta_{i}$ is the vector of fixed effects (including the classificatory effect of CG and the effect of age of the animal at the time of evaluation as linear and quadratic covariates), $a_{i}$ is the vector of direct additive genetic effects and $e_{i}$ a vector of random residual effects, where X and Z are the incidence matrices relating the elements in $l_{i}$ to the vectors $\beta_{i}$ and $a_{i}$ , respectively. We assumed that $a_{i}∼N\left(0,A\sigma_{a}^{2}\right)$ and $e_{i}∼N\left(0,I\sigma_{e}^{2}\right)$ , where A is the numerator relationship matrix, I is an identity matrix, $\sigma_{a}^{2}$ and $\sigma_{e}^{2}$ are the additive and residual genetic variance components, respectively. As $\sigma_{e}^{2}$ is not estimable for binary models (Gianola and Foulley, 1983), the parameterization $\sigma_{e}^{2}=1$ was used (Sorensen and Gianola, 2002). *A priori*, the residual covariance was considered equal to zero, and remained at zero after being estimated by the software.

The relationship between the score for the j th animal ( $y_{j}$ ) and its liability ( $l_{ij}$ ) for FLM can be represented by:

$${\left\{y\right.}_{j}=0,if\;l_{ij}\leq t;\left.y_{j}=1,if\;l_{ij}>t\right\}$$

in which: $y_{j}$ is the score for the j th animal, t corresponds to threshold that define, on the underlying scale, the mutually exclusive categories of FLM (0 or 1) (Gianola and Foulley, 1983; Vargas et al., 2018). The prior distributions for the variance components and the fixed and random effects are assumed to be flat. A 500,000-cycle Markov chain was generated in the YW-FLM two-trait analysis with an initial discard period of 50,000 cycles and sampling interval of 50 cycles. The convergence of the chains was evaluated by Geweke (1992) and Heidelberger and Welch (1983) tests, using the packages CODA (Plummer et al., 2006) and BOA (Smith, 2007) in language R version 4.1.2 (R Development Core Team, 2017), in addition to visual inspection.

### 2.3 Genotyping and quality control

Genotyping was completed from 6,624 animals using the Illumina BovineHD Genotyping BeadChip (HD; Illumina, Inc., San Diego, CA, United States), which included 612,174 SNP markers, and 28,155 animals genotyped with medium density panels (20–90 k SNP). All animals belong to the Nellore Alliance population (GenSys, 2019) and had their own performance record and/or progeny evaluated for FLM. The FImpute v3 (Sargolzaei et al., 2014) software was used for genotype imputation from the lower density panels to the HD SNP chip and marker coordinates were mapped according to the genomic positions provided by new bovine genome assembly ARS-UCD1.2 (Hu et al., 2022).

Quality control (QC) of the genotypic and pedigree datasets was performed using QCF90 software (Masuda et al., 2019) and the following criteria were used for the exclusion of SNP markers: *p*-value < 10–5 for the Hardy-Weinberg equilibrium test; minor allele frequency (MAF) < 0.02; and a call rate <90%.

### 2.4 Genome-wide association study

The GWAS results were reported as the proportion of the variance explained by non-overlapping genomic windows of 20 adjacent SNP (Zhang et al., 2016; Hay and Roberts, 2018; Oliveira et al., 2019; Zhuang et al., 2020). The phenotypes of FLM were used as dependent variables in a single-trait threshold animal model. SNP effects were estimated by the weighted single-step genomic BLUP (WssGBLUP) method with two iterations, as proposed by Wang et al. (2012), using the programs of BLUPF90 family (Misztal et al., 2014). As in Vargas et al. (2018) the solutions ( $\hat{u}$ ) were obtained as a function of the genomic estimated breeding values (GEBVs) through the equation:

$$\hat{u}=DZ^{\prime }{\left[ZDZ^{\prime }\right]}^{-1}{\hat{a}}_{g}$$

where D is a diagonal matrix of weights for SNP variances; Z is a matrix relating genotypes of each locus; and ${\hat{a}}_{g}$ is the vector of GEBVs of genotyped animals. The vector $\hat{u}$ and the matrix D were iteratively recalculated through the S2 method (looping for step 2) of the algorithm created by Wang et al. (2012). The results from the second iteration were used, because they generally provide greater accuracy for genomic predictions (Vallejo et al., 2016; Lourenco et al., 2020) and marker effects (Wang et al., 2012; Irano et al., 2016; Melo et al., 2016).

The POSTGSF90 software was used to estimate the proportion of variance explained by SNP effects and the criterion used to identify potentially important genomic regions was the sum of the variance in non-overlapping windows of 20 adjacent SNPs, approximately 130 Kb (average density of one SNP per 6.5 kb). Windows that explained greater than 1% of the genetic variance were selected for gene annotation, conducted using R Version 4.1.2 (R Development Core Team, 2017) and the R package GALLO (Fonseca et al., 2020). The .gtf file Bos_taurus.ARS-UCD1.2.104.gtf, corresponding to the bovine reference map ARS-UCD1.2 (Hu et al., 2022) was used for gene annotation.

As LD values (r^2^) > 0.08 are observed up to 400 Kb of distance between marker pairs for the Nellore breed, with higher linkage disequilibrium estimates (r^2^ > 0.15) observed in the first 100 Kb (Pérez O’Brien et al., 2014), we evaluated the presence of genes in adjacent 100 Kb windows upstream and downstream of the main windows (average annotation interval of 330 Kb). We also visually inspected the windows with the Genome Data Viewer (NCBI, 2022), in case candidate gene annotation failed to detect related genes located near the coordinates.

### 2.5 Gene prioritization analysis

The candidate gene prioritization was conducted using GUILDify and ToppGene (Chen, Bardes, et al., 2009; Guney et al., 2014). GUILDify relies on biological databases that predominantly focus on model organisms such as humans, mice and flies, which have been extensively studied and have well-characterized genetic and molecular interaction data. The limited availability of genomic and molecular data for *Bos indicus* restricts its suitability for interaction networks on GUILDify. Thus, a “trained list” of the top 100 genes associated with relevant trait keywords, including, “mobility aplomb,” “feet,” “foot angle,” “leg,” “rear legs,” “leg conformation,” “leg structure,” “conformational structure,” “feet quality,” “leg quality,” “articular cartilage,” “osteogenic differentiation,” “lameness resistance,” and “hoof health” was created using GUILDify and a species-specific (*Homo sapiens*) interaction network to identify orthologous genes between humans and cattle Then, the ToppGene Suite was used to perform a prioritization analysis comparing the functional information shared between the list of “trained” genes and the list of “test” genes (genes located within windows that explained more than 1% of the genetic variance). Gene Ontology terms (molecular function, biological process, and cellular component), human and mouse phenotypes, metabolic pathways, PubMed publications, coexpression patterns and diseases were used to retrieve the functional information from genes in training and testing lists (Fonseca et al., 2018). We selected, as prioritized, the genes on the “test” list with the same functional profile as the genes of “trained” list, based on a multiple correction false discovery rate of 5% (*p*-value ≤ 10–3) (Chen, Bardes, et al., 2009).

## 3 Results

**FIGURE 1:**
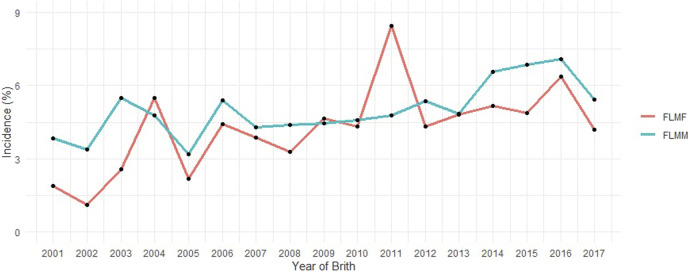
Incidence of feet and legs malformation (FLM) at yearling in males (FLMM) and females (FLMF) by year of birth.

The incidence value (approximately 5%) for FLM after consistency checks and preliminary editing described above should be highlighted (Table 1). The pedigree file contained 468,302 animals and the incidence of FLM has increased in the most recent years for both sexes (Figure 1) in the population studied. There was a statistically significant difference by Student’s *t*-test (*p* < 0.05) in the annual incidence averages between genders. Males (average incidence = 4.98%) were more affected than females (average incidence = 4.23%) by FLM in the period evaluated. After QC, 461,057 SNP remained. All genotyped samples had a call rate per individual greater than 0.9. Genotypes from 12,537 animals passed QC and were used in the GWAS analyses.

**TABLE 1:** Descriptive structure of Nellore data used in the yearling weight-feet and legs malformation (YW-FLM) two-trait analysis.

Number of phenotypic records	295,031
Number of sires with progeny with phenotypic records	3,853
Mean age of animals at the time of evaluation (in days)	526
Mean weight of animals at measurement (in kg)	297.98
Standard deviation of the weight of the animals at measurement (in kg)	53.51
Number of contemporary groups	6,628
Minimum number of animals per contemporary group	10
Maximum number of animals per contemporary group	533
Number of males	177,130
Number of females	117,901
FLM incidence (%)	5.34

**FIGURE 2:**
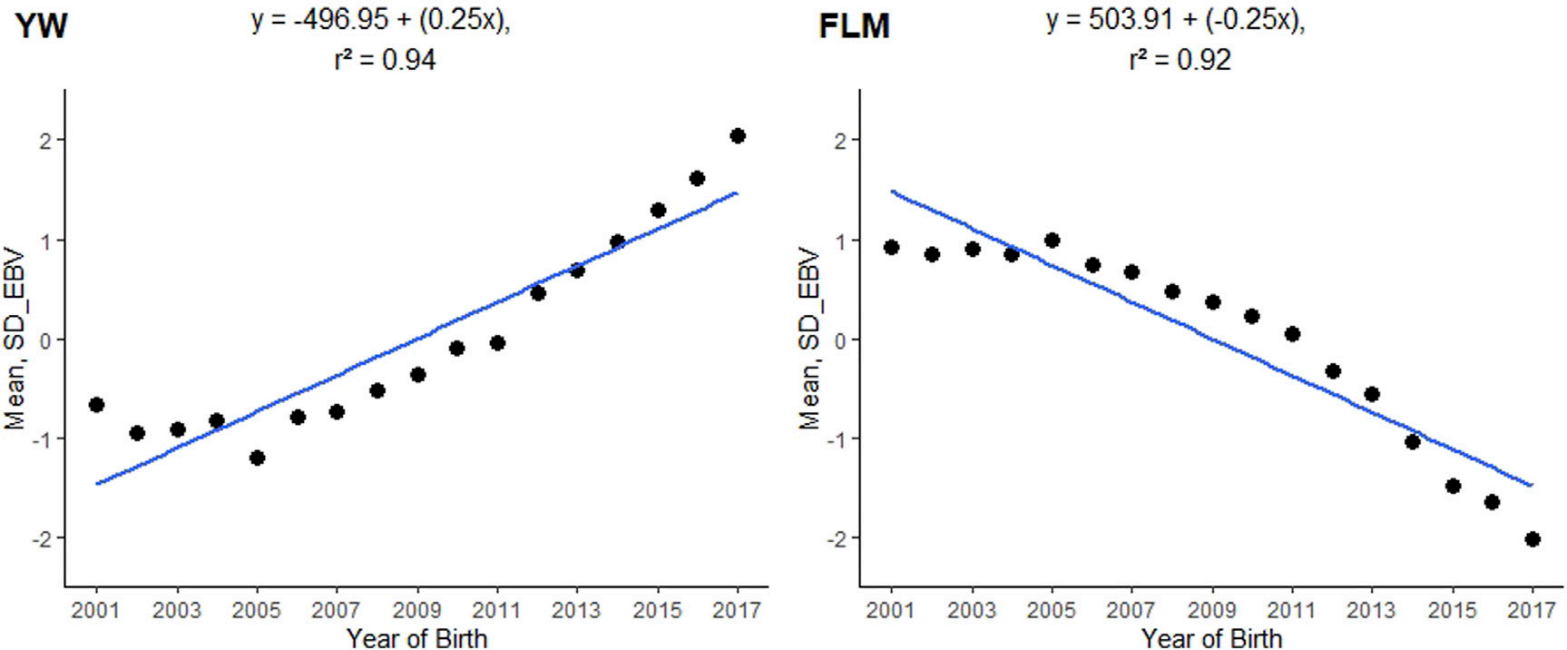
Genetic trends of posterior means of breeding values by year of birth for yearling weight (YW) and feet and legs malformation (FLM) evaluated at yearling in Nellore cattle; SD_EBV = a posterior mean of the estimated breeding value expressed in units of genetic standard deviation.

The posterior mean of the heritability estimates (L95%; U95%) for FLM was of moderate magnitude (0.1856). In single-trait analyses for comparison purposes, the mean heritability estimated for FLM (L95%; U95%) was 0.17 (0.13; 0.20). The linear trait (YW) had a high heritability value (0.4718) (Table 2), while the posterior mean estimate of the genetic correlation between FLM and YW was −0.2311 (±0.0076). The annual genetic trends for YW and FLM were 0.25 and −0.25 units of genetic standard deviation, respectively (Figure 2).

**TABLE 2:** Estimates of additive ( $\sigma_{a}^{2}$ ) and residual ( $\sigma_{e}^{2}$ ) genetic variance components, covariance ( σ ), heritability ( $h^{2}$ ) and genetic correlation ( r ) obtained in YW-FLM two-trait analysis in Nellore cattle.

Parameter^a^	$\sigma_{a}^{2}$ _(YW)_	σ _(YW,FLM)_	$\sigma_{a}^{2}$ _(FLM)_	$\sigma_{e}^{2}$ _(YW)_	$\sigma_{e}^{2}$ _(FLM)_	$h^{2}$ _(YW)_	$h^{2}$ _(FLM)_	r _(YW,FLM)_
Mean	341.64	−4.3829	0.2304	382.50	1.0074	0.4718	0.1856	−0.2311
L95%	331.10	−4.6750	0.1718	374.00	1.0000	0.4590	0.1458	−0.2460
U95%	352.40	−4.0940	0.2925	390.80	1.0150	0.4848	0.2250	−0.2164
Median	341.50	−4.3820	0.2298	382.50	1.0070	0.4717	0.1858	−0.2311
Mode	341.00	−4.3500	0.2350	383.00	1.0070	0.4725	0.1850	−0.2325
SD	5.5063	0.1457	0.0305	4.3181	0.0037	0.0067	0.0200	0.0076
Z-Geweke	0.8291	0.1150	0.5754	0.3386	0.0964	0.6027	0.5233	0.1478
ESS	1583.9	316.8	51.5	1571.5	9000.0	1574.1	68.0	360.7

^a^ Posterior mean and respective lower (L95%) and upper (U95%) limits for the 95% credibility interval for yearling weight (YW) and feet and legs malformation (FLM), posterior Median, posterior Mode, Standard Deviation (SD), Z-Geweke (p-value), Effective Sample Size (ESS).

**FIGURE 3:**
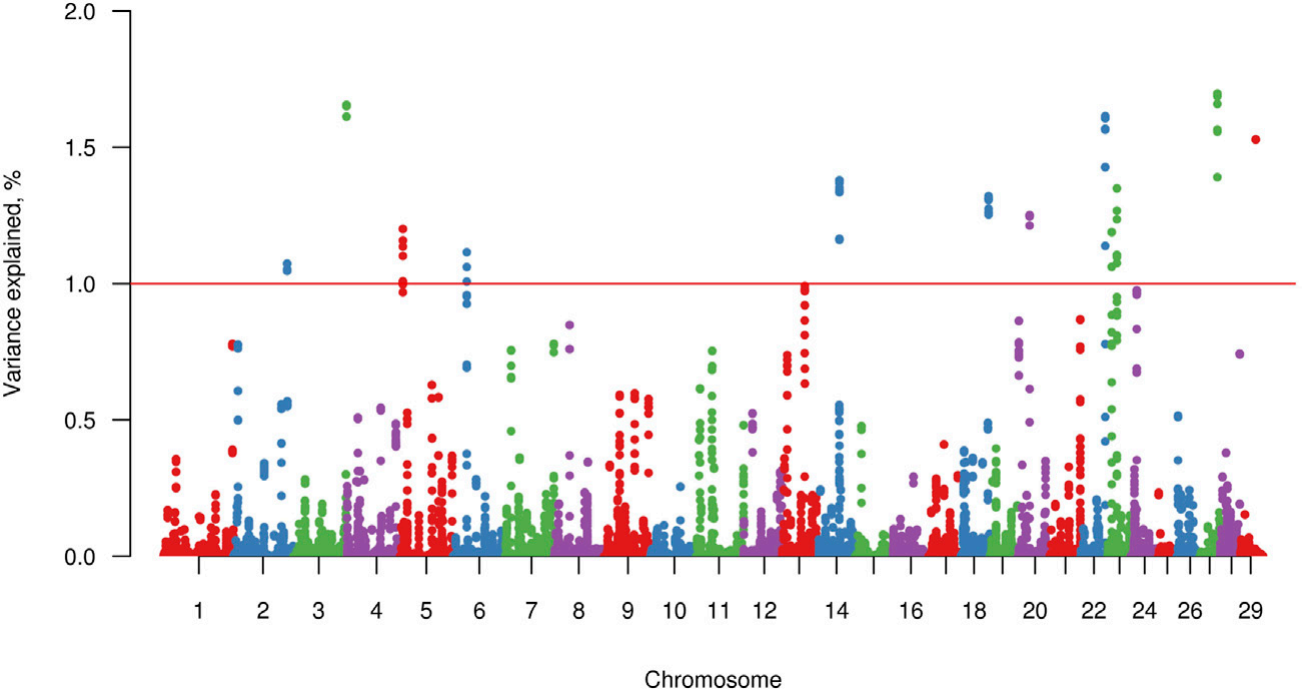
Manhattan plot for percentage of variance explained by non-overlapping windows of 20 adjacent SNP for feet and legs malformation obtained by WssGBLUP method. The red line indicates the 1% variance threshold explained by the windows.

Twelve windows, two of these located on chromosome BTA23 and one on each of chromosomes BTA2, BTA3, BTA5, BTA6, BTA14, BTA18, BTA20, BTA22, BTA27, and BTA29, explained >1% of the genetic variance of FLM. Together, these windows explained 16.37% of the total genetic variance of FLM (Figure 3). Considering 100 Kb upstream and downstream non-overlapping windows of 20 consecutive SNPs, 61 positional candidate genes for FLM were identified. The window containing the most FLM candidate genes (17 genes) was in BTA3, and the window explaining the highest proportion of variance (1.7%) was in BTA27. The four candidate genes (*EXT1*, *ITGA1*, *ATG7*, and *PPARD*) prioritized by GUILDify and ToppGene analysis were in four windows, which together explained 5.43% of the total genetic variance. Additionally, skeletal development-related genes *SCUBE3* and *SHOX* that were not prioritized in the ToppGene analysis were identified by visual inspection in the Genome Data Viewer (NCBI, 2022). We also identified 24 genes (starting from *LOC*) for which no associated sign gene was present (Table 3). There were not any orthologous genes for these *LOC* that could be interesting to discuss in the FLM context.

**TABLE 3:** Annotated genes within the windows of 20 adjacent SNP that explained more than 1% of genetic variance for FLM in Nellore cattle.

Window region			Var (%)	Gene names
BTA^a^	Start	End		
27	39,428,405	39,525,725	1.70	*LRRC3B*
3	119,525,250	119,674,756	1.66	*CSF2RA*, *LOC100336476*, *IL3RA*, *LOC112446045*, *LOC107131293*, *LOC112446046*, *P2RY8*, *LOC112445918*, *AKAP17A*, *LOC112445919*, *LOC112446047*, *LOC617467*, *LOC526047*, *COPS9*, *OTOS*, *LOC782275*, and *LOC112446048*
22	55,269,065	55,321,257	1.61	*HRH1* and *ATG7* ^b^
29	35,078,320	35,112,359	1.53	*NTM* and *LOC112444927*
14	46,405,542	46,506,899	1.38	*EXT* ^b^
23	20,946,123	21,032,829	1.35	*LOC107131722* and *PTCHD4*
18	62,415,280	62,498,670	1.32	*EPS8L1*, *RDH13*, *LOC100848752*, *GP6, NLRP2*, *LOC100336589*, *LOC100852077*, *LOC112442414*, *LOC100301263*, *NCR1*, *FCAR*, *KIR2DL5A*, *KIR3DL1*, and *KIR3DS1*
20	26,181,870	26,332,917	1.25	*ITGA1* ^b^, *PELO*, and *LOC112443001*
5	6,318,396	6,420,053	1.20	*ZDHHC17*, *CSRP2*, *LOC112446899*, and *E2F7*
23	9,228,154	9,312,557	1.19	*TCP11*, *SCUBE3* ^c^, *LOC101907009*, *ZNF76*, *DEF6*, and *PPARD* ^b^
6	30,757,118	30,803,603	1.11	*ATOH1*, *GRID2*, *TRNAE-UUC*, and *LOC112447051*
2	121,473,182	121,525,407	1.07	*LOC112442276*, *CLDN12*, *SHOX* ^c^, *LOC112442278*, and *MIR2887-1*

^a^ BTA, *Bos taurus* autosome.

^b^ Prioritized candidate genes identified by GUILDify and ToppGene analysis, considering 100 Kb upstream and downstream of the window.

^c^ Prioritized candidate genes identified by window inspection in the Ensembl genome browser.

## 4 Discussion

New-born animals must participate in their own nutrition and locomotion to survive. Thus, they are endowed with a set of muscles that grew during prenatal life in such a way that they play their part in ensuring survival. The new-born animal is able to walk and to stuck because “early developing” muscles of the distal parts of the limbs are well developed at birth (Berg and Butterfield, 1974). During their development, however, the musculoskeletal system is influenced by genetic factors that, if expressed, alter the animal’s posture. In addition, environmental factors such as overfeeding, the availability and distribution of food and water, and the topography of grazing environments can also contribute to persistent postural issues (Vargas et al., 2018).

Differences in growth curves and body weight composition between the sexes can be explained by hormonal differences such as androgenic effects (e.g., testosterone) (Young and Bass, 1984; Mangwiro et al., 2013). In the Nellore breed, there is little difference between male growth and female growth before weaning, but this difference becomes more pronounced with age (Malhado et al., 2009; Arruda et al., 2018). This could explain the observed differences in the incidence of FLM in males and females (Figure 1). The observed overall incidence rate (5.34%) was higher than that reported by Vargas et al. (2017) (4.5%), who used a subsample from a single Nellore Alliance program with approximately one-third of the observations used in this study. The increase in the incidence of FLM in recent years in the same population may be influenced by the subjectivity of assessment and should be observed with caution. As field technicians gain experience over the years, they tend to place higher discerning demands on the evaluation. “Knock-kneed” (when the knee joints lie inside this line), “bow-legged” (when the knee joints lie outside this line) and “straight-legged” (high angulation of the tibiotarsal joint) (Vargas et al., 2018) are examples of malformation patterns that can be best rated by an experienced technician.

The magnitude of heritability estimated for the FLM depends on the rating assigned to the animal at the time of assessment and may be directly related to the quality of the recording set measured for the classification of the trait (Vargas et al., 2017). Furthermore, using YW as an “anchor,” in a two-trait analysis, may help capture a greater proportion of additional variability. Studies in dairy cows have shown heritability estimates for leg and foot conformation traits to range from 0.03 to 0.22 (Berry et al., 2004; Chapinal et al., 2013; Häggman and Juga, 2013). In the Nellore breed, Passafaro et al. (2013) and Vargas et al. (2018), Vargas et al. (2017) obtained moderate to high heritability estimates of the FLM, as estimated in this study, which indicating the potential for improvement through selection.

The assessment of the genetic correlation between YW and FLM (−0.2311) was considered favourable, since in FLM, the lowest taxonomic rank (score 0) indicated that animals did not have feet and legs defects, whereas in YW animals with stronger expression of the trait are required. For example, in the population studied, animals that had difficulty walking due to congenital deformities of the legs and feet (score 1) tended to gain less weight. Consistent with the results of this study, Vargas et al. (2017) estimated the genetic correlation between YW and FLM to be 0.39, on a scale of 1–5 (ranging from least ideal to most ideal). This suggests that animals with better legs and feet tend to be heavier. In addition to YW-related responses, which are part of the selection index, estimated genetic trends from FLM suggest that strategies cull problem animals promote favorable genetic changes and facilitate the genetic progression of the trait in the study population.

A literature search, such as that carried out by Vargas et al. (2018), is an important step in the process of identifying functional candidate genes mapped around candidate markers. However, if a greater number of positional candidate genes are identified, the literature review may become unfeasible (Fonseca et al., 2018). The strategy of automating gene prioritization analysis (Martins de Carvalho et al., 2020; Sweett et al., 2020) speeds up the identification of genes related to the regulation of biological processes associated with the phenotype. Combining GUILDify and ToppGene in a single analysis is a novel approach in the functional prioritization literature (Kominakis et al., 2017). Fonseca et al. (2018) highlight the importance of combining tools and functional information from multiple sources (and species) to perform a better selection of functional candidate genes and further explore GWAS results. ToppGene does not use keywords to select genes, instead, the software uses the similarities between the functional patterns of the genes presented in the candidate gene list and the trained gene list. Therefore, the prioritized genes presented can be interpreted as a statistical measure of how much the functional profile of each candidate gene is like the entire functional profile of the trained list (GUILDify) (Martins de Carvalho et al., 2020).

With the aim of finding genes that are closely associated with the phenotype and are critical for its development and maintenance, the present GWAS allowed to find genes reported in the literature with traits related to the feet and legs conformation in dairy and beef cattle populations. For example, the specific deletion in the muscle tissue of the *ATG7* gene (BTA22) in mice that resulted in profound muscle atrophy and age-dependent decrease in strength (Masiero et al., 2009). The *ATG7* gene (BTA22) encodes an E1-like essential activating enzyme for autophagy and cytoplasmic transport to the vacuole (Gao et al., 2013). The autophagy flow is important to preserve muscle mass and maintain the integrity of the myofiber. In beef cattle, increased *ATG7* gene expression was correlated with skeletal muscle growth and body weight during the fattening period, regardless of the muscle evaluated (Nakanishi et al., 2019).

The *EXT1* gene (BTA14) encodes a glycosyltransferase responsible for heparan sulfate (HS) polymerization, related to fibroblast growth factor (Nadanaka et al., 2008). The interaction between growth factors and their receptors is regulated by the amount of HS. A defect in HS biosynthesis due to somatic mutations in *EXT1* can cause a localized break in the negative feedback loop that regulates chondrocyte proliferation and maturation, allowing premature differentiation and therefore aberrant bone growth (Duncan et al., 2001).

Another prioritized candidate gene, *ITGA1* (BTA20), encodes a subunit of the cell-surface receptor integrin α1β1, which acts in the regulation of hepatic glucose and lipid metabolism under conditions of overnutrition *in vivo*. On a high fat diet, mice with the *ITGA1* gene inactivated show severe hepatic insulin resistance and decreased hepatic fat accumulation (Williams et al., 2015). In Korean Hanwoo cattle, the *ITGA1* gene was significantly associated with intramuscular fat deposition (Lee et al., 2013; de las Heras-Saldana et al., 2020). Weight gain accelerated by increased fat deposition in young animals can cause stress on ligaments, tendons and joints, promoting gene expression patterns that predispose to alterations in the normal conformation of feet and legs.

The same association can be made for the *PPARD* gene (BTA23), the most abundant form of Peroxisome Proliferator-Activated Receptor (*PPAR*) family in skeletal muscle (Brennan et al., 2009). A key role for the *PPARD* gene in controlling the biological processes that drive ruminal epithelial cell development in new-born calves was suggested by Naeem et al. (2012). Its increased expression after improved diet suggests an important role in promoting the use of long-chain fatty acids as substrates for oxidation in cell membranes during differentiation in ruminal development. In dairy cows, the *PPARD* gene is involved in muscle fatty acid transport and oxidation during early lactation, and muscle fat replacement from days 3 to 30 of lactation (Schäff et al., 2013). Studies have shown that the gene is highly expressed in the intestinal epithelium, keratinocytes, and liver, consistent with an important biological role in these tissues and influenced by the onset lactation and the type of lipids that are supplied (Girroir et al., 2008; Akbar et al., 2013).

The *SCUBE3* (BTA23) and *SHOX* (BTA2) genes, identified by visual inspection of significant windows of the FLM, have relevant roles in bone formation. The *SCUBE3* gene acts as a BMP2/BMP4 (bone morphogenetic protein) coreceptor and positively regulates signalling possibly enhancing the specific interaction between BMP and BMP type I receptors (Lin et al., 2021). Malfunction of the *SCUBE3* gene has been linked to problems such as craniofacial and dental defects, reduced body size, and defective endochondral bone growth in mice, and osteosarcoma in humans (Liang et al., 2015). The *SCUBE3* and *PPARD* genes are in the same window of *BTA23*, 79 kb apart. The presence of antagonistic causal mutations in these two genes, that is, an increased expression in *PPARD* influencing accelerated growth and a malfunction in *SCUBE3* promoting bone defects suggests a possible biological mechanism for the genetic correlations observed between FLM and YW. However, it is important to emphasize that this information is specific to the mentioned genes and their relation to specific traits. Additional studies and deeper analyses would be needed to confirm and fully understand the role of these genes and their interactions in relation to the mentioned traits.

Rafati et al. (2016) found a complete association between the *SHOX* gene deletion and the skeletal atavism of the Shetland pony. It is a genetic disorder characterized by abnormal growth of the ulna and fibula, which elongate the carpal and tarsal joints, respectively. This can lead to abnormal bone structure and restricted movement. The *SHOX* gene is also associated with skeletal defects such as Léri-Weill dyschondrosteosis (Benito-Sanz et al., 2012), short stature, and limb deformities in humans (Jorge et al., 2007; Chen, Wildhardt, et al., 2009; Raudsepp et al., 2012).

## 5 Conclusion

Overall, this study provides valuable information on the genetic basis of feet and leg malformations in Nellore cattle, with important implications for both animal welfare and agricultural productivity. Estimates of additive genetic variability and heritability for this trait suggest that selection may reduce the incidence of this problem. The phenotypic incidence shows the importance of reinforcing this selection, although the estimated trend curve shows a relatively favorable genetic gain over the study period. Furthermore, the genetic association of yearling weight with feet and legs malformations may have implications for selection criteria used in breeding programs. The current GWAS was able to identify chromosomal regions associated with feet and legs malformation in Nellore cattle. The roles of prioritized candidate genes in skeletal muscle development (*ATG7*), aberrant bone growth (*EXT1*), lipid metabolism (*ITGA1*, *PPARD*), intramuscular fat deposition (*ITGA1*), and skeletogenesis (*SCUBE3*, *SHOX*) underscore the importance of these genes in malformation incidence. The identification of genes associated with foot and leg malformations allows selective breeding of animals that are less likely to develop these conditions, leading to healthier and more productive herds. The identified genetic markers could facilitate the development of genetic tests that can identify carriers of these mutations, allowing breeders to make more informed breeding decisions that reduce the incidence of malformations in future generations.

## Data Availability

The data analyzed in this study was obtained from GenSys Associated Consultants, the following licenses/restrictions apply: access is restricted to protect confidential or proprietary information. Requests to access these datasets should be directed to Haroldo Neves, gensys.haroldo@gmail.com.
